# Assessment of mHealth Interventions: Need for New Studies, Methods, and Guidelines for Study Designs

**DOI:** 10.2196/21874

**Published:** 2020-11-18

**Authors:** Roxana Ologeanu-Taddei

**Affiliations:** 1 TBS Business School Toulouse France

**Keywords:** eHealth, mHealth, usability, management, survey, trust, guidelines, evaluation

## Abstract

This viewpoint argues that the clinical effects of mobile health (mHealth) interventions depends on the acceptance and adoption of these interventions and their mediators, such as usability of the mHealth software, software performance and features, training and motivation of patients and health care professionals to participate in the experience, or characteristics of the intervention (eg, personalized feedback).

## Background

In the past years, designs of research studies in medical journals have been formalized according to the reporting guidelines of academic associations, international consortia, and publishers, which enable publications of clinical trials, systematic reviews, and meta-analyses. The use of mobile health (mHealth) technologies is expected to increase, creating new paths for health care delivery. However, there are no specific guidelines to enable researchers to design and present their studies and results on this topic, except for the existing guidelines on randomized clinical trials (RCTs), which can be used for certain assessments of mHealth interventions.

## Measures of Clinical Effects Require Opening the Black Box of Information Technology

There is no doubt that RCTs are useful to assess the clinical outcomes and effectiveness of mHealth. In this context, regulatory bodies such as the US Food and Drug Administration (FDA) [1] and European regulations have recently updated the requirements for clinical proofs of mHealth solutions. Nevertheless, the focus on RCT methods leads to the black boxing of mHealth interventions [2], which means that the technology is considered as a homogeneous device or as a pharmaceutical substance. This view misses the main characteristic of an mHealth intervention (and overall, that of information technology [IT])—the embeddedness of data and clinical processes (as reflected in the guidelines for diagnosis and personalized monitoring). Therefore, assessing the clinical effect of an mHealth intervention should disentangle the effect of a new process of personalized monitoring, the effect of the ubiquitous access enabled by mobile devices, and the comprehension and adoption of clinical guidelines implemented into the application. In addition, mHealth solutions may differ from one another because of their different designs of these processes.

## Need of Standard Guidelines to Assess Technology and Mediators of Outcomes

Moreover, the clinical effect of mHealth solutions depends on the acceptance and adoption of these solutions and their mediators, such as usability of the mHealth software, software performance and features, training and motivation of patients and health care professionals to participate in the experience, or characteristics of the intervention (eg, personalized feedback). For example, a clinical effect such as survival benefits for patients with cancer who use a surveillance mHealth app depends on the acceptance and adoption of the app, which can be influenced by the usability of the app and patients’ prior experience in using mobile apps, motivation or trust in IT, or other alternative mediators contributing to the main reported outcome; this influences are often neglected by RCTs.

Settings of mHealth interventions must be carefully described and assessed in hypothesis-generating studies [3], such as observational studies and case reports. These studies can identify specific moderators and mediators that state for whom and under what conditions the health intervention works [3]. Moderators may identify population groups with possible causal mechanisms or courses of illness. The mHealth mediators identify possible causal mechanisms, meaning causal links between the intervention and the outcome, through which the intervention may achieve its effects [3]. As a next step, these moderators and mediators should be considered as stratification variables in forthcoming RCTs focused on hypothesis testing. Otherwise, RCTs are likely to be based on weak assumptions rather than empirical evidence.

## Beyond Effectiveness: Risk Assessment

In addition, RCTs need to be complemented by other clinical trials and case reports to assess safety risks [4] and unintended consequences of mHealth. The acknowledgment of these risks is at the core of the updated regulations for medical devices, which include software and mHealth. However, in most cases, assessments of mHealth safety risks are conducted separately [5], for example, in feasibility or usability studies, which use different methods of varying rigor and do not generate cumulative knowledge. In addition, case reports on the adoption stages of mHealth solutions should be inspired by engineering methods (eg, fault tree analysis rather than pharmacovigilance studies). Moreover, relevant and cumulative knowledge can be gathered if publications on the issues of usability and user acceptance are presented not only in health informatics journals but also in major medical journals, as these issues cause clinical effects. For example, the Journal of the American Medical Association (JAMA) Network advises authors to use the guidelines of the EQUATOR (Enhancing the QUAlity and Transparency Of health Research) network [6], which include guidelines related to “economic studies.” Similar initiatives should be undertaken for studies concerning mHealth. In recent years, cumulative knowledge has been gathered on the risks associated with poor usability of health IT [7]. In line with this literature, a step forward was taken only for mHealth solutions, which are qualified as medical devices [8]. However, even for those applications, national and international regulations (ie, CE marking in Europe or FDA regulations) and harmonized standards (ie, EN 62366 advised for CE marking in Europe) strengthened the requirements for premarket certifications but did not standardize a threshold for usability or technical performance. We must recognize that recommending the minimum required sample size (eg, 15 users identified by user profile numbers) makes an improvement to the summative usability assessment method [9]. It is also necessary to assess user profiles accurately. These user profiles may be related to health care occupations (eg, clinical secretaries, physicians, or nurses), different health departments (eg, infectious diseases or pediatrics), and social or demographic variables. In addition, several other characteristics (eg, computer literacy, prior experience of using mobile apps, and users’ engagement or trust in IT) may mediate or moderate mHealth effects and therefore could be taken into account to refine user profiles. Further research is needed on these moderators and mediators to use them as criteria for construction of user profiles. Although such research can be complex and costly, it is relevant and useful.

## Need of Studies in Implementation and Adoption Stages

The implementation of mHealth solutions (beyond pilots) in the market and their user adoption stages introduce new contextual and technological factors, such as low technical performance, lack of interoperability with existing systems, or misfit with existing clinical practices. Pilot studies often benefit from specific resources—both financial and human—and from high motivation of the patients and health care professionals involved. These factors may be missing in the latter stages of implementation and adoption, influencing the outcome achievement or introducing risks to patient safety. Although these challenges cannot be experimentally controlled, they may be assessed cautiously in rigorous qualitative and statistical studies. In addition, the moderators and mediators of mHealth interventions, such as engagement levels [10], should be investigated more systematically.

We have to mention that the EQUATOR network published guidelines for the reporting of mobile phone–based health interventions [11]. Formed by the World Health Organization’s panel of experts, these guidelines are useful to improve the transparency and harmonization of the reporting of mHealth, enabling comparisons and meta-reviews. These criteria include usability/content testing and user feedback. Nevertheless, a new step must be taken toward formulating guidelines on study designs and methods of the assessment of user feedback on mHealth interventions.

## Moving Forward: Formal Guidelines for Study Designs on Real-World Usage

The evaluation of moderators and mediators in pilots should be followed by larger surveys and follow-ups during the whole life cycle of the mHealth technology [5]. The protocols of these studies should be inspired by the rigor of protocols of clinical investigations while considering the relevant factors specific to mHealth. Open trials, observational studies, and case reports should be conducted to measure mediators beyond a specific clinical setting (the variables and low sample size of which could introduce serious bias). In addition, anecdotal reports and qualitative studies should use common frameworks, which will facilitate systematic reviews and afford transferability. The knowledge generated can thus inform policy decisions.

Moreover, anecdotal reports of suspected adverse reactions related to the use of mHealth (along with reports of health IT–related adverse events) should be encouraged and published in medical journals, as mHealth can induce specific errors related to the use of technology. These issues have been emphasized as crucial for more than 20 years, during which numerous studies have shown that bad informatics can have fatal consequences [5]. If the new European regulations on medical devices (which include mHealth solutions) require real-life, postmarket, clinical, and risk assessment follow-ups of these devices, new methods and frameworks should be elaborated with scientific rigor and inspired by different academic disciplines, such as engineering and social sciences. Building on the guidelines for research presentations and knowledge from medical informatics and risk engineering [12], it is time to make rigorous evaluations and formalize guidelines for research presentations, enabling evidence-based mHealth interventions.

## Abbreviations

EQUATOR: Enhancing the QUAlity and Transparency Of health Research
FDA: Food and Drug Administration
IT: informational technology
JAMA: Journal of the American Medical Association
mHealth: mobile health
RCT: randomized clinical trial
